# Supplementary material for: lncRNA read-through regulates the BX-C insulator Fub-1
Source: eLife. 2023 Aug 10;12:e84711. doi: 10.7554/eLife.84711 (PMC10497285; doi:10.7554/eLife.84711)
Supplement: Supplementary file 1. — (a) The sequence of chimeric DNA fragments used for Fab-7 replacement experiments. (b) Sequences of Fub-1 sense smFISH probes covering 1983 bp region in bxd domain. (c) Sequences of Fub-1 antisense smFISH probes covering 1463 bp region in bxd domain. [file elife-84711-supp1.docx]

**Supplementary file 1a. The sequence of chimeric DNA fragments used for *Fab-7* replacement experiments:**

**The sequence of *HS2^505R^-HS1^248R^-CTCF^x4^* fragment containing multimerized binding sites for dCTCF (CTCF×4). The binding sites are marked in red. *SphI* restriction sites are underscored.**

CAGCTTATAAACGTAGAAATGTATGGAAAATCTTATTGTGCATGATATAATTATAAGATTTTTAAGTAAAAATCTTTTCATATTAAAGAAAGAACAATTTTGTAAAAATGAATTGAAGTTCTCGTGCCATTTCTTACATTTTGCGCCGACGGGCTATCAAAATGAAAAGAGTGAGAGGAACCTGAAGAATCGGAATCGAGGAGAATGAGTGCCTCGAAATACTCACTCAAACGAAAAGGCCAAGCCCATTTTCTTGCTGGTGGTGGTGATGAAATGCTGGATTTTTTCTGGCTCGTTCAACCACCGCGCCACATTTCCAGTTTCGACTTGCTCCGGCGCAGACGTCGCTTAAGGGGCGGCGGATGAGCGATATCTGACAAAAAGGCCAAAGACAAACAAAAGCAGAGGCTAGCAAGCCAAAAAAAAATATTGTAAATATTTCGAAATTAGATCAGTGAAGATTTGAAAAATATAAAAGAACCGGAAGAAGATAAATCTACAAAGCTACGAGATATAAATTCGTTTAAATATTAACGGAAAATAGGTGGGAAACGCATATTTCTTACGTCAGATGGCGGCACTGAGTT*GCATGC*(***SphI***)GC**GCGCCATCTAGCGGCATG**TGACGCGTTCC**GAATCCATAGATGGCGCA**TGAACGCGTA**TGGCCGACAGATGGCTCG**TCTCGAGTC**AAGATGTCGCTCTCCGAC**AGGAT*GCA****TGC***(***SphI***)**GCCATCTATGGATTC**GGTTTTCATAGTTTTAGAGTCATCTTGTCGGTCCAGTTTTAGAGCCACTGTTACTCCTTTGAAGTTGTTCTATGTTACATAACATAAATATGTTAAATGAAATGTTAGAATTAAAAAAGGTGGGCGAATATTTTCGCTTTTAGCT

**The sequence of *HS2^505R^-SV-HS1^248R^* fragment containing *SV40* terminator. *SV40* sequence is marked in red, polyadenylation sites are bolded and underscored.**

CGTAGAAATGTATGGAAAATCTTATTGTGCATGATATAATTATAAGATTTTTAAGTAAAAATCTTTTCATATTAAAGAAAGAACAATTTTGTAAAAATGAATTGAAGTTCTCGTGCCATTTCTTACATTTTGCGCCGACGGGCTATCAAAATGAAAAGAGTGAGAGGAACCTGAAGAATCGGAATCGAGGAGAATGAGTGCCTCGAAATACTCACTCAAACGAAAAGGCCAAGCCCATTTTCTTGCTGGTGGTGGTGATGAAATGCTGGATTTTTTCTGGCTCGTTCAACCACCGCGCCACATTTCCAGTTTCGACTTGCTCCGGCGCAGACGTCGCTTAAGGGGCGGCGGATGAGCGATATCTGACAAAAAGGCCAAAGACAAACAAAAGCAGAGGCTAGCAAGCCAAAAAAAAATATTGTAAATATTTCGAAATTAGATCAGTGAAGATTTGAAAAATATAAAAGAACCGGAAGAAGATAAATCTACAAAGCGAATTCGATCATAATCAGCCATACCACATTTGTAGAGGTTTTACTTGCTTTAAAAAACCTCCCACACCTCCCCCTGAACCTGAAACATAAAATGAATGCAATTGTTGTTGTTAACTTGTTTATTGCAGCTTATAATGGTTACA***AATAAA***GCAATAGCATCACAAATTTCACA***AATAAA***GCATTTTTTTCACTGCATTCTAGTTGTGGTTTGTCCAAACTCATCAATGTATCTTAAGATCTGCTAAAAGCGAAAATATTCGCCCACCTTTTTTAATTCTAACATTTCATTTAACATATTTATGTTATGTAACATAGAACAACTTCAAAGGAGTAACAGTGGCTCTAAAACTGGACCGACAAGATGACTCTAAAACTATGAAAACCGAATCCATAGATGGCGCATGCAACTCAGTGCCGCCATCTGACGTAAGAAATATGCGTTTCCCACCTATTTTCCGTTAATATTTAAACGAATTTATATCTCGTAG

**The sequence of *HS2^505R^-mSV-HS1^248R^* fragment containing *SV40* terminator with mutated polyadenylation sites. *SV40* sequence is marked in red; polyadenylation sites were replaced with XhoI and MluI restriction sites (bolded and underscored).**

CGTAGAAATGTATGGAAAATCTTATTGTGCATGATATAATTATAAGATTTTTAAGTAAAAATCTTTTCATATTAAAGAAAGAACAATTTTGTAAAAATGAATTGAAGTTCTCGTGCCATTTCTTACATTTTGCGCCGACGGGCTATCAAAATGAAAAGAGTGAGAGGAACCTGAAGAATCGGAATCGAGGAGAATGAGTGCCTCGAAATACTCACTCAAACGAAAAGGCCAAGCCCATTTTCTTGCTGGTGGTGGTGATGAAATGCTGGATTTTTTCTGGCTCGTTCAACCACCGCGCCACATTTCCAGTTTCGACTTGCTCCGGCGCAGACGTCGCTTAAGGGGCGGCGGATGAGCGATATCTGACAAAAAGGCCAAAGACAAACAAAAGCAGAGGCTAGCAAGCCAAAAAAAAATATTGTAAATATTTCGAAATTAGATCAGTGAAGATTTGAAAAATATAAAAGAACCGGAAGAAGATAAATCTACAAAGCGAATTCGATCATAATCAGCCATACCACATTTGTAGAGGTTTTACTTGCTTTAAAAAACCTCCCACACCTCCCCCTGAACCTGAAACATAAAATGAATGCAATTGTTGTTGTTAACTTGTTTATTGCAGCTTATAATGGTTACA***CTCGAG****(XhoI)*GCAATAGCATCACAAATTTCACA***ACGCGT****(MluI)*GCATTTTTTTCACTGCATTCTAGTTGTGGTTTGTCCAAACTCATCAATGTATCTTAAGATCTGCTAAAAGCGAAAATATTCGCCCACCTTTTTTAATTCTAACATTTCATTTAACATATTTATGTTATGTAACATAGAACAACTTCAAAGGAGTAACAGTGGCTCTAAAACTGGACCGACAAGATGACTCTAAAACTATGAAAACCGAATCCATAGATGGCGCATGCAACTCAGTGCCGCCATCTGACGTAAGAAATATGCGTTTCCCACCTATTTTCCGTTAATATTTAAACGAATTTATATCTCGTAG

**Supplementary file 1b. Sequences of *Fub-1 sense* smFISH probes covering 1983-bp region in *bxd* domain.**

| **Oligonucleotide sequences**  **Probe set name: *Fub-1 sense*** | |
| --- | --- |
|  | **SEQUENCE (5' to 3')** |
| 1 | tttgtcagatatcgctcatc |
| 2 | ctgcttttgtttgtctttgg |
| 3 | tttttttttggcttgctagc |
| 4 | cttcttccggttcttttata |
| 5 | tctcgtagctttgtagattt |
| 6 | ccacctattttccgttaata |
| 7 | ccgccatctgacgtaagaaa |
| 8 | aaccgaatccatagatggcg |
| 9 | actggaccgacaagatgact |
| 10 | aaggagtaacagtggctcta |
| 11 | aaaatattcgcccacctttt |
| 12 | agttttcattggggttaagt |
| 13 | ggttggttggaaaggaagta |
| 14 | tgggcaaataggcagtatca |
| 15 | cagtcgatcgtaaaccggag |
| 16 | cgttgacccctaaacaagtt |
| 17 | gtctttgattggcaatatgc |
| 18 | tggcaagtcggggaaattga |
| 19 | tggaatcgtttgcactgcaa |
| 20 | atcttgatgctttgattgcc |
| 21 | ttgttgtcttaaccgcattt |
| 22 | ccctaacgatgctcaaagtg |
| 23 | cagttttgagctggaatcgt |
| 24 | gatgattttcatgttttccc |
| 25 | tgcgaatgtcgaaagcgcat |
| 26 | tttagccgttttagcggatg |
| 27 | ctgattgcgattggtgttct |
| 28 | gcaactcgaagccaacaact |
| 29 | ttagtttattttctgcccat |
| 30 | tgttatggcttggatctttc |
| 31 | cggcagccaaatgaaaagca |
| 32 | ccaaagaaaccagttggtgc |
| 33 | gtgctggagatgggaagata |
| 34 | tgatgatgggtcgagtttca |
| 35 | cactttaccgagtcttggag |
| 36 | ctgcttttattggtttgcac |
| 37 | ggtgatatacttatttcgca |
| 38 | tcactaatacacgttccagt |
| 39 | acaattccgtcctgaatagt |
| 40 | cactgagaggcccaaaacaa |
| 41 | tggtgctcaattgcatgatg |
| 42 | catggatatagggagcagga |
| 43 | gcatcagcttagatgaagtt |
| 44 | gaaccgtttctgtatcggtg |
| 45 | aattgtcgaccgtagagcga |
| 46 | acggaaccaagccaaactga |
| 47 | cgattattttacggccacta |
| 48 | gtcgcaataaccacgacatt |

**Supplementary file 1c. Sequences of *Fub-1 antisense* smFISH probes covering 1463-bp region in *bxd* domain.**

| **Oligonucleotide sequences**  **Probe set name: *Fub-1 antisense*** | |
| --- | --- |
|  | **SEQUENCE (5' to 3')** |
| 1 | tcggtcacttcatatgttgt |
| 2 | agtcatcttttctcggtttg |
| 3 | gcaacatctgccaattactg |
| 4 | gcaacacggcaaaagcatcg |
| 5 | aattaagttgtgccgcacac |
| 6 | tgccaacacgctctatgaat |
| 7 | atatcatggtgttcaccgta |
| 8 | ttatgaagtcgtcaagcggc |
| 9 | ccaactgctcatacatcaca |
| 10 | caagccatcgtgaagaggat |
| 11 | cgggccaatttgaaacactt |
| 12 | tgacgactttattcgtgcga |
| 13 | gaactgcgcacttcatttga |
| 14 | atggaccaacatcgatggtg |
| 15 | gattcaactatgtctcgggg |
| 16 | ttttgtggtgctgggtaaat |
| 17 | gcagctatctttaagcagtt |
| 18 | tcataatcaccacgagacgc |
| 19 | acttatctgcccttttttac |
| 20 | ctatttttacggatcccttg |
| 21 | atgaatgtgtgtgcgagtgt |
| 22 | aatggcaggactcgtaactc |
| 23 | gctgcctgctattttaattt |
| 24 | ggaattgtatggcctttgac |
| 25 | caacatgaccattttgtccg |
| 26 | tatctgtgagaatgcggcat |
| 27 | tatgatatggcttcatcggg |
| 28 | tataatgcccaagcgacttt |
| 29 | tcaacagggctgctcgaaaa |
| 30 | accctttcaaatgcaaagca |
| 31 | caaaatggggtggccaagat |
| 32 | gcctaaacaggctggaaaga |
| 33 | cctacaactgggtcaagttt |
